# Is the treatment with pulses of methylprednisolone safe?

**DOI:** 10.1186/1756-6614-6-S2-A6

**Published:** 2013-04-05

**Authors:** Tomasz Bednarczuk, Piotr Miśkiewicz

**Affiliations:** 1 Department of Endocrinology and Internal Medicine, Medical University of Warsaw, Warsaw, Poland

## 

High dose intravenous (iv) methylprednisolone (MP) pulse therapy is a highly effective immunosuppressive treatment used in various inflammatory and autoimmune diseases. Active, moderate to severe Graves' orbitopathy (GO) and neuropathy due to GO are indications for this treatment. Treatment with MP iv in GO is more effective than with oral glucocorticoids and better tolerated. In patients receiving MP iv there are significantly less adverse events, related to exogenous Cushing syndrome, such as weight gain, hypertension, "Cushingoid" feature. The European Group on Graves' Orbitopathy (EUGOGO) advises treatment with high doses iv MP pulses in patients with active, moderate to severe GO and neuropathy due to GO. However, recent studies indicate that this treatment may be associated with an increased risk of severe and fatal side effects due to the cardiovascular complications (acute cardiac arrest, pulmonary embolism, myocardial infarction, acute heart failure, stroke) or acute liver failure. The cardiovascular side effects after treatment with high doses of MP iv are probably due to the influence on hemostasis and cardiovascular system (increased blood pressure, influence on myocardium contractility and heart insufficiency). Treatment with high doses iv MP pulses can influence glucose metabolism, bone metabolism, psychosis. The exact pathomechanism of this side effects and safety profile of the treatment are so far unknown. It is very important to evaluate all risk factors, indications and contraindications before treatment with high doses iv MP pulses. All patients should conduct regular follow up during this treatment.

